# Prophylactic Vancomycin in the Primary Prevention of *Clostridium difficile* in Allogeneic Stem Cell Transplant

**DOI:** 10.1111/tid.70025

**Published:** 2025-03-18

**Authors:** Brendon Fusco, Jasmine Tomita‐Barber, Natale Mazzaferro, Anne Tyno, Nicole McEntee, Patricia Greenberg, Roger Strair, Dale Schaar, Dennis Cooper

**Affiliations:** ^1^ Montefiore Einstein Comprehensive Cancer Center Albert Einstein College of Medicine New York USA; ^2^ Department of Internal Medicine Rutgers Robert Wood Johnson School of Medicine New Brunswick New Jersey USA; ^3^ Department of Biostatistics and Epidemiology Rutgers School of Public Health Piscataway Township New Jersey USA; ^4^ Robert Wood Johnson School of Medicine Rutgers Cancer Institute of New Jersey New Brunswick New Jersey USA

**Keywords:** allogeneic stem cell transplant, *Clostridium difficile*, primary prevention, prophylaxis, vancomycin

## Abstract

**Background:**

*Clostridium difficile* (*C. difficile*) infection is a frequent complication following hematopoietic stem cell transplant, contributing to increased morbidity in this population. Despite current infection prevention strategies, rates among posttransplant patients at some centers remain high. Oral vancomycin is a safe and well‐tolerated antibiotic that may be a potential adjunct to prevent *C. difficile*.

**Objectives:**

To evaluate the effectiveness of oral vancomycin in preventing *C. difficile* among allogeneic stem cell transplant patients and assess other posttransplant outcomes.

**Study Design:**

A retrospective cohort study was conducted comparing the rate of *C. difficile* following allogeneic transplant in patients who received oral vancomycin versus no prophylaxis during hospitalization. The primary outcome was the development of *C. difficile* infection within the first 100 days following transplant, defined as a positive stool toxin assay or PCR. Secondary outcomes included hospital length of stay, hospital‐acquired infections, overall mortality, graft‐versus‐host disease (GVHD), and rehospitalization.

**Results:**

Among the 202 patients, one case of *C. difficile* occurred in the prophylaxis group (1/71, 1.4%) compared to 31 cases in the unexposed group (31/131, 23.6%). Patients who received prophylaxis were significantly less likely to develop *C. difficile* infection during the study period (OR 0.046, *p* = 0.003). No differences were observed between groups in hospital‐acquired infections, mortality, incidence of acute GVHD, and rehospitalization.

**Conclusion:**

Prophylactic vancomycin was associated with a marked reduction in *C. difficile* infection in allogeneic transplant patients. Despite no significant impact on other clinical outcomes, there was a significant reduction in symptomatic *C. difficile* infection. Further prospective studies are warranted.

## Graphical abstract

Prophylactic oral vancomycin was associated with reduced *Clostridium difficile* infection in allogeneic stem cell transplant patients. No differences in hospital‐acquired infections, mortality, GVHD, or rehospitalization were observed. It is a low‐cost, effective preventative strategy, though further prospective studies are needed to confirm safety and efficacy.

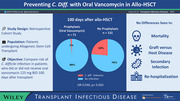

**Abbreviations**
ALL: acute lymphoblastic leukemia
AOR: adjusted odds ratio
*C. difficile*
: *Clostridium difficile*
CI: confidence interval
GVHD: graft‐versus‐host disease
MDS: myelodysplastic syndrome
PCR: polymerase chain reaction
VRE: vancomycin‐resistant enterococci

## 1 Introduction

*Clostridium difficile* (*C. difficile*) infection represents a frequent infectious complication among stem cell transplant patients, with an estimated incidence of 9%–31% [1, 2]. While most *C. difficile* infections in these patients are mild to moderate in severity, infection has been associated with increased mortality in some studies [3, 4]. Multiple factors contribute to an increased risk of *C. difficile* infection in allogeneic transplant patients, including conditioning regimen‐induced gut dysbiosis, frequent broad‐spectrum antibiotic exposure, and prolonged neutropenia [1, 5]. For example, the broad application of fluoroquinolones for prophylaxis is a major risk factor [6]. For these reasons, rates of *C. difficile* infection are high in the hematopoietic stem cell transplant population, particularly in the first 100 days after transplant; one epidemiologic retrospective study found that up to 78% of cases occurred during the first 100 days following transplant with a median time to infection of 33 days [3].

The strategies for *C. difficile* prevention at most institutions include antimicrobial stewardship practices, barrier precautions, and sporicidal agents in environmental cleaning [7, 8]. Despite these interventions resulting in a reduction in incidence [7, 8, 9], infection rates among some institutions remain high. For this reason, there is ongoing interest in preventing *C. difficile* infection in hematopoietic stem cell transplant patients. Oral vancomycin is a well‐tolerated, safe, and effective treatment for *C. difficile* infection with a desirable safety profile [10]. In a retrospective study that included 145 patients, Ganetsky et al. found that oral vancomycin during transplant eliminated *C. difficile* infection [11]. In a meta‐analysis study, Maraolo et al. also found that oral vancomycin was effective in preventing primary and secondary *C. difficile* infections in patients treated with systemic antibiotics [12]. However, we are not aware of any studies of primary prophylaxis in transplant centers where there has been a consistently high rate of *C. difficile* infection.

In this study, we examined the effectiveness of oral, low‐dose vancomycin in preventing *C. difficile* in this population and potential impact on other important posttransplant outcomes such as length of stay, bacterial infections, overall mortality, graft‐versus‐host disease (GVHD), and rehospitalization.

## 2 Methods

In 2019, the Department of Transplant and Cellular Therapy at Robert Wood Johnson University Hospital implemented oral vancomycin, 125 mg twice daily, to all patients admitted to the Bone Marrow Transplant unit for hematopoietic stem cell transplantation. Patients were started on oral vancomycin from admission to discharge unless there was a known contraindication to vancomycin, such as a prior allergic reaction. Following transplantation and discharge, all patients readmitted to the hospital were started on oral vancomycin at the same dose throughout admission. This intervention began as a quality improvement project to address a perennial *C. difficile* infection rate of approximately 20% on our unit despite standardized antimicrobial stewards and environmental precautions. We conducted a retrospective cohort study of allogeneic transplant patients to examine the effectiveness of oral vancomycin in preventing *C. difficile* infection in comparison to allogeneic transplant patients who did not receive prophylaxis before the intervention. As many autologous transplant patients were not regularly seen in the 100 days following transplant, they were excluded from this analysis. A small number of umbilical cord blood transplant patients were also excluded. This study compared allogeneic transplant patients exposed to oral vancomycin at prophylactic doses from 2019 to 2021 with those without exposure between 2016 and 2019.

The study's primary outcome was the development of *C. difficile* infection during the first 100 days (study period) following an allogeneic transplant. Testing for *C. difficile* infection followed a standard department protocol. Before the pandemic, patients admitted to the unit were tested for *C. difficile* infection if they had at least three unformed bowel movements classified as Bristol Stool Scale 6 or 7. Testing was conducted only if they had not undergone *C. difficile* testing in the preceding 7 days, had no exposure to oral contrast or initiated on tube feeds in the previous 24 h, and had not received laxatives in the preceding 72 h. With the start of the COVID‐19 pandemic in 2020, the protocol was updated to promote earlier detection of *C. difficile* infection. Patients were tested if they had at least one unformed bowel movement classified as Bristol Stool Scale 6 or 7 during hospital Days 1 through 3. All specimens were tested by Robert Wood Johnson University Hospital's Microbiology Laboratory with the *C. Diff* Quik Chek Complete rapid membrane immunoassay, simultaneously detecting glutamate dehydrogenase antigen and Toxins A and B, with reflex to polymerase chain reaction (PCR) when a positive glutamate dehydrogenase but negative toxin antigen test resulted (Sensitivity 92.3%, Specificity 97.7%) [13]. All patients throughout the study period were admitted to the same hospital unit, and standard hand washing and barrier precautions were implemented when a patient was suspected of *C. difficile* infection.

Secondary outcomes of interest included hospital length of stay, development of other infections, including vancomycin‐resistant enterococci (VRE) infection, all‐cause mortality, GVHD, hospital readmission, and readmission for infection during the study period. Infectious disease data wa collected systematically by examining all blood, central line, and urine cultures during the study period. Untreated asymptomatic bacteriuria was excluded from the data. Patients were considered readmitted for infection if the primary reason for their presentation was a bacterial infection, including bacteremia/sepsis, pneumonia, abscess, and catheter‐associated infections. If not the primary reason for presentation, bacterial infections were still included in the data. Because oral vancomycin could change the microbiome and the latter may play a role in GVHD [14], hospital discharge summaries and biopsies were reviewed during the study period to assess the incidence of acute GVHD. Patient characteristics of interest included age, gender, type of malignancy, donor match, degree of HLA match (defined as 9/10, 10/10, or other/haploidentical), levofloxacin exposure (explicitly defined as at least 2 consecutive days of levofloxacin 500 mg once daily for bacterial prophylaxis in high‐risk neutropenia), and conditioning intensity (categorized as myeloablative or reduced intensity).

### 2.1 Statistical Methods

Baseline characteristics and outcomes of interest were compared using a Wilcoxon rank‐sum test for continuous measures and either an *X* ^2^ test or Fisher's exact test for categorical measures. In addition to bivariate comparisons, both a multivariable stepwise logistic regression for binary outcomes (such as whether the patient had a positive *C. difficile* test) and multivariable linear regression for continuous outcomes (e.g., hospital length of stay) were used to examine study outcomes adjusted for other demographic and clinical factors. All variables found to have a significant *p* value in the bivariate analyses, defined as < 0.05, were included in the final regression models, including age, gender, malignancy, conditioning intensity, degree of match, and use of levofloxacin. All continuous variables were tested for the assumption of normality using the Shapiro–Wilk test, and a visual quantile–quantile plot inspection deemed them nonparametric. All analyses were conducted using SAS statistical software (version 9.4) (SAS Institute, Cary, North Carolina).

## 3 Results

### 3.1 Patient Characteristics

A total of 202 patients were included in the study. Seventy‐one patients received oral vancomycin prophylaxis from 2019 to 2021, while 131 received no prophylaxis from 2016 to 2019. Patient characteristics are included in Table 1. The median age of those exposed to vancomycin was 59, whereas the median age of the unexposed group was 55 (*p* = 0.07). There were no significant differences in gender, donor source, or degree of match between groups. There was a significantly higher proportion of acute lymphoblastic leukemia (ALL) patients in the unexposed group compared to the exposed group (18.32% vs. 7.04%, *p* = 0.047), and patients in the exposed group had a higher proportion of myeloablative conditioning (73.24% vs. 56.49%, *p* = 0.02). No patients in the unexposed group received oral levofloxacin, whereas 53.52% of patients in the exposure group received levofloxacin.

**TABLE 1:** Patient characteristics.

Variables	Overall *n* = 202	No vancomycin *n* = 131	Vancomycin *n* = 71	*p* value
**Age in years at screening**				0.07^a^
Range	20–75	20–75	20–71	
Median [IQR]	57 [46, 63]	55 [44, 63]	59 [51, 65]	
Mean (SD)	53.59 (13.28)	52.32 (13.77)	55.94 (12.08)	
**Gender (%)**				0.97^b^
Male	122 (60.4)	79 (60.31)	43 (60.56)	
Female	80 (39.6)	52 (39.69)	28 (39.44)	
**Donor (%)**				0.61^b^
Haplo	99 (49.01)	62 (47.33)	37 (52.11)	
Related	57 (28.22)	40 (30.53)	17 (23.94)	
Unrelated	46 (22.77)	29 (22.14)	17 (23.94)	
**Degree (%)**				0.06^c^
Other	57 (28.22)	40 (30.53)	17 (23.94)	
9/10	14 (6.93)	5 (3.82)	9 (12.68)	
10/10	131 (64.85)	86 (65.65)	45 (63.38)	
**Malignancy (%)**				**0.047** ^b^
ALL	29 (14.36)	24 (18.32)	5 (7.04)	
AML	110 (54.46)	70 (53.44)	40 (56.34)	
CML	6 (2.97)	3 (2.29)	3 (4.23)	
MDS	22 (10.89)	9 (6.87)	13 (18.31)	
Myelofibrosis	10 (4.95)	7 (5.34)	3 (4.23)	
Other	25 (12.38)	18 (13.74)	7 (9.86)	
**Conditioning intensity (%)**				**0.02** ^b^
Myeloablative	126 (62.83)	74 (56.49)	52 (73.24)	
Reduced intensity conditioning (RIC)	76 (37.62)	57 (43.51)	19 (26.76)	
**Levofloxacin (%)**				**< 0.0001** ^c^
Yes	38 (18.81)	0 (0)	38 (53.52)	
No	164 (81.19)	131 (100)	33 (46.48)	

*Note*: Other malignancies include: peripheral T‐cell lymphoma, primary cutaneous gamma‐delta T‐cell lymphoma, classical Hodgkin's lymphoma, adult T‐cell leukemia/lymphoma, diffuse large B‐cell lymphoma, marginal zone lymphoma, and mantle cell lymphoma.

Abbreviations: ALL: acute lymphoblastic leukemia, AML: acute myeloblastic leukemia, CML: chronic myelogenous leukemia, IQR: interquartile range, MDS: myelodysplastic syndrome, SD: standard deviation.

^a^ Wilcoxon rank‐sum test

^b^ chi‐square

^c^ Fisher's exact test

### 3.2 Primary Outcome

There was one case of *C. difficile* infection in patients who received oral vancomycin prophylaxis (1/71 patients, 1.4%), compared with 31/131 (23.6%) cases in the unexposed group (Table 2, *p* < 0.001). Total 15.3% of patients in the unexposed group tested positive within the first 30 days, 6.1% in the following 30 days, and 2.3% in the remaining 40 days. In the exposed group, one case occurred on Day 96. As shown in Table 3, patients who received oral vancomycin prophylaxis were 21.7 times less likely to develop *C. difficile* infection during the study period (unadjusted OR 0.046, confidence interval [CI] 95% 0.006–0.346, *p* = 0.003). When adjusting for age, gender, malignancy, conditioning intensity, degree of match, and use of levofloxacin, patients who received oral vancomycin were 6.85 times less likely to develop *C. difficile* (adjusted OR 0.146, CI 95% 0.018–1.174, *p* = 0.07). A stepwise logistical model was employed to account for the low event rate in the exposure group. This model found that none of the mentioned adjusted clinical factors significantly contributed to the result and returned the same OR as the unadjusted Cox model.

**TABLE 2:** Bivariate outcomes.

Primary outcome	Overall *n* = 202	No vancomycin *n* = 131	Vancomycin *n* = 71	*p* value
***C. difficile* diagnosis and timeframe (%)**				**< 0.0001** ^c^
No diagnosis of *C. difficile*	170 (84.16)	100 (76.34)	70 (98.59)	
30 days	20 (9.90)	20 (15.27)	0 (0)	
60 days	8 (3.96)	8 (6.11)	0 (0)	
100 days	4 (1.98)	3 (2.29)	1 (1.41)	
**Secondary Outcome**				
**Length of stay**				0.06^a^
Range	15–139	18–132	15–139	
Median [IQR]	25 [23, 30]	24 [22, 30]	26 [24, 30]	
Mean (SD)	31.74 (21.05)	31.89 (21.83)	31.46 (19.68)	
**Infection during hospitalization (%)**				0.25^b^
Yes	82 (40.59)	57 (43.51)	25 (35.21)	
No	120 (59.41)	74 (56.49)	46 (64.79)	
**Death (%)**				0.84^b^
Yes	16 (7.92)	10 (7.63)	6 (8.45)	
No	186 (92.08)	121 (92.37)	65 (91.55)	
**Graft‐versus‐host disease (%)**				0.59^b^
Yes	17 (8.42)	10 (7.63)	7 (9.86)	
No	185 (91.58)	121 (92.37)	64 (90.14)	
**Hospital readmission (%)**				0.46^b^
Yes	59 (29.21)	36 (27.48)	23 (32.39)	
No	143 (70.79)	95 (72.52)	48 (67.61)	
**Readmission for infection (%)**				0.62^b^
Yes	26 (12.87)	18 (13.74)	8 (11.27)	
No	176 (87.13)	113 (86.26)	63 (88.73)	

Abbreviations: IQR, interquartile range SD, standard deviation.

^a^ Wilcoxon rank‐sum test

^b^ chi‐square

^c^ Fisher's exact test

**TABLE 3:** Multivariate regression analysis.

Outcome	Vancomycin exposure	OR (95% CI)	*p* value	AOR (95% CI)^a^	*p* value
***C. difficile* infection**	No	—	—	—	—
	Yes	0.046 (0.006, 0.346)	**0.003**	0.146 (0.018, 1.173)	0.07
**Stepwise model: *C. difficile* infection**	No			—	—
	Yes			0.046 (0.006, 0.346)	**0.003**
**Hospital infection**	No			—	—
	Yes	0.706 (0.388, 1.282)	0.25	1.535 (0.967, 3.497)	0.31
**Death**	No	—	—	—	—
	Yes	1.117 (0.388, 3.211)	0.84	0.957 (0.181, 5.065)	0.96
**Graft‐versus‐host disease**	No	—	—	—	—
	Yes	1.324 (0.481, 3.642)	0.59	0.296 (0.030, 2.902)	0.3
**Rehospitalization**	No	—	—	—	—
	Yes	1.264 (0.675, 2.369)	0.46	0.870 (0.350, 2.160)	0.76

*Note*: Logistical and stepwise regression of unadjusted and adjusted outcomes.

Abbreviations: AOR: adjusted odds ratio, CI: confidence interval, OR: odds ratio.

^a^ Adjusted for age, gender, malignancy, conditioning intensity, degree of match, and levofloxacin.

### 3.3 Secondary Outcomes

There were no differences in the development of hospital‐acquired infections (AOR 1.535, CI 95% 0.97–3.5, *p* = 0.31), all‐cause mortality (AOR 0.95, CI 95% 0.18–5.1, *p* = 0.96), GVHD (AOR 0.296, CI 95% 0.03–2.9, *p* = 0.3), or rehospitalization (AOR 0.87, CI 95% 0.35–2.16, *p* = 0.76) during the study period (Table 3). No deaths were attributable to *C. difficile* infection, and there were no cases of VRE in all 202 study patients, as shown in Table 2. The median length of stay for patients who received prophylactic vancomycin was 26 days, with a range of 15–139 days, compared with 24 days for the unexposed group, with a range of 18–132 days (*p* = 0.06, Table 2). In addition, there was no significant difference between groups in the rehospitalization rate for infection following discharge during the study period (11.3% vs. 13.7%, *p* = 0.62).

## 4 Discussion

In the present retrospective study, there appeared to be a marked reduction in the incidence of *C. difficile infection* with the use of prophylactic oral vancomycin. One out of 71 patients who received vancomycin developed *C. difficile*, compared with 23.7% of 131 patients who did not receive vancomycin. This is in keeping with Ganetsky et al., who also demonstrated a significant reduction in infection in patients who received vancomycin prophylaxis, and a recent meta‐analysis by Maraolo et al. [11, 12]. No cases of VRE were found, a described complication of parenteral exposure to vancomycin but with unknown risk after oral vancomycin. Notably, the one patient who developed *C. difficile* infection in the exposure group tested positive on Day 96 rather than closer to the time of transplantation and there were no clinical symptoms based on provider documentation. Outside of Ganetsky et al., prior studies primarily focus on the use of vancomycin in the secondary prevention of *C. difficile* infection [15, 16]. One prospective randomized clinical trial also found once‐daily oral fidaxomicin to reduce infection rates among these patients [17]. However, this study suffered from a lower‐than‐expected infection rate in the nonintervention arm and a high dropout rate. Fidaxomicin is more expensive than oral vancomycin; at most institutions, it remains the second‐line treatment for less severe *C. difficile* infection due to cost and lack of formulary adoption [18]. Oral vancomycin has a high tolerability, excellent safety profile, and is less expensive to implement.

Despite the reduction in *C. difficile* infection rates, no significant difference was observed in overall mortality, length of hospital stay, development of hospital‐acquired infections, and rehospitalization following discharge between groups. The lack of impact on mortality, hospitalization, and readmission probably reflects the mild nature of most *C. difficile* infections. This is not to underestimate the psychosocial consequences of isolation and symptom burden of *C. difficile* diarrhea, especially following an allogeneic stem cell transplant. Although this study did not demonstrate a difference in secondary outcomes, the prevention of *C. difficile* infection has important implications for hospital resource utilization and also makes management of diarrhea, a common complication of allogeneic transplant, more straightforward including the use of anti‐diarrhea therapy. Reducing *C. difficile* infection can also decrease the use of personal protective equipment, cleaning supplies, and the staffing and time required for room decontamination.

There were imbalances between the different cohorts. For example, patients who received vancomycin prophylaxis were older (median age of 59 vs. 55), more likely to receive myeloablative conditioning (73% vs. 56%), and had markedly higher use of levofloxacin for the primary prophylaxis of bacterial infections in high‐risk neutropenia (53% vs. none). However, these factors would be expected to increase the risk of *C. difficile* infection [1, 5]. Although the implementation of levofloxacin for high‐risk neutropenia at our institution coincided closely with the implementation of prophylactic oral vancomycin, this decision was made independently. The adoption of prophylactic levofloxacin at this time was based on emerging data supporting its effectiveness in reducing morbidity and preventing bacterial infections in this population. It is now considered a standard of care [19, 20]. Nevertheless, we cannot exclude the possibility that less use of systemic parenteral antibiotics due to levofloxacin prophylaxis might have been associated with decreased *C. difficile* infections. Clearly, a randomized study with both populations either receiving levofloxacin or not would be important to help exclude confounding factors. Importantly, we did not see an increase in VRE infections, similar to the meta‐analysis of Maraolo et al.

### 4.1 Limitations

The present study has several limitations, including its retrospective design. For example, in 2019, with the onset of the COVID pandemic, our Infectious Disease Control department observed stronger adherence to hand sanitizer use and dramatically decreased total number of patient visitors. The hospital also implemented the standard use of sporicidal cleaning agents in room decontamination. These factors could have decreased the spread of *C. difficile* between patients who are yet undiagnosed, and barrier precautions could have led to decreased rates of *C. difficile* infection in the exposure group. However, equipment used for contact precautions did not change in the study period. In addition, *C. difficile* infection rates on general hospital floors did not change during the pandemic, reflecting the overall spread of *C. difficile* pre‐ and post‐pandemic was not significantly different or affected by these practices.

As noted above, the implementation of levofloxacin for high‐risk neutropenia prophylaxis closely coincided with the implementation of vancomycin. As levofloxacin prophylaxis was associated with a non‐statistically significant decrease in bacteremia, there may have been less use of parenteral antibiotics in the prophylaxis group, leading to less VRE. Clearly, a prospective clinical trial in which both groups have comparable prophylaxis is required.

An additional limitation of our study is in sample size and statistical analyses. Due to the low event rate in the exposure group, it was difficult for multivariate models to adjust for the mentioned clinical factors. In addition, the low number of patients in the exposure group limits the study's power to detect differences in secondary outcomes. While we employed a stepwise model to investigate which clinical factors significantly contributed to this change in odds, the model only showed that no clinical factors had any significant impact and returned the same result as the unadjusted OR. Overall, the significantly reduced rate of *C. difficile* infection is apparent, especially given that the single event in the exposure group occurred on Day 96 and was not clinically significant.

## 5 Conclusion

The present study demonstrates a significant association between the prophylactic use of oral vancomycin and a decreased rate of *C. difficile* infection in patients undergoing allogeneic stem cell transplantation. At centers with a high prevalence of *C. difficile*, oral vancomycin is a low‐cost intervention that could be considered to reduce infection rates. Although this study did not show an impact on select clinical outcomes, such as overall mortality or length of hospital stay, the potential benefits of decreased symptom burden and reduced hospital resource utilization should not be overlooked. Further prospective trials with close attention to safety outcomes are warranted to confirm these findings and further examine any deleterious effects of vancomycin.

## Author Contributions

**Brendon Fusco**: investigation, writing – original draft, methodology, validation, writing – review and editing, conceptualization, project administration, data curation, resources, formal analysis. **Jasmine Tomita‐Barber**: resources, project administration. **Natale Mazzaferro**: formal analysis, software, writing – review and editing. **Anne Tyno**: conceptualization. **Nicole McEntee**: conceptualization. **Patricia Greenberg**: formal analysis, software. **Roger Strair**: conceptualization, investigation. **Dale Schaar**: conceptualization, resources. **Dennis Cooper**: conceptualization, investigation, supervision, writing – review and editing, project administration, resources.

## Ethics Statement

Ethical approval for this study was obtained from Rutgers Institutional Review Board, Human Research Protection Program (Approval # Pro2023000856).

## Conflicts of Interest

The authors declare no conflicts of interest.

## Data Availability

The data that support the findings of this study are available on request from the corresponding author. The data are not publicly available due to privacy or ethical restrictions.
